# Effect of a 4-Week Internet-Delivered Mindfulness-Based Cancer Recovery Intervention on the Symptom Burden and Quality of Life of Patients With Breast Cancer: Randomized Controlled Trial

**DOI:** 10.2196/40059

**Published:** 2022-11-22

**Authors:** Luyao Wang, Xing Chen, Yueyang Peng, Kun Zhang, Jun Ma, Lin Xu, Zixuan Liu, Li Liu, Yang Luo, Can Gu

**Affiliations:** 1 Xiangya School of Nursing Central South University Changsha China; 2 Hunan Key Laboratory of Oral Health Research & Xiangya Stomatological Hospital Central South University Changsha China; 3 School of Nursing Xinjiang Medical University Urumqi China

**Keywords:** mindfulness-based cancer recovery, mindfulness-based intervention, cancer-related symptom, quality of life, breast cancer, internet-delivered intervention, mobile phone

## Abstract

**Background:**

Mindfulness-based interventions (MBIs) can improve the symptoms and psychological well-being of patients with breast cancer. However, standard MBIs are an 8-week program delivered face-to-face, which may be inconvenient for patients with cancer. Many attempts have been made to adapt MBIs to increase their accessibility for patients with cancer while maintaining their therapeutic components and efficacy.

**Objective:**

This study aimed to investigate the effectiveness of a 4-week internet-delivered mindfulness-based cancer recovery (iMBCR) program in reducing symptom burden and enhancing the health-related quality of life (HRQoL) of patients with breast cancer.

**Methods:**

A total of 103 postoperative patients with breast cancer (stages 0 to IV) were randomly assigned to an iMBCR group (4-week iMBCR; n=51, 49.5%) or a control group (usual care and 4-week program of health education information; n=52, 50.5%). The study outcomes included symptom burden and HRQoL, as measured by the MD Anderson Symptom Inventory and the Functional Assessment of Cancer Therapy-Breast scale. All data were collected at baseline (T0), after the intervention (T1), and at 1-month follow-up (T2). Data analysis followed the intention-to-treat principle. Linear mixed models were used to assess the effects over time of the iMBCR program.

**Results:**

Participants in the iMBCR group had significantly larger decreases in symptom burden than those in the control group at T1 (mean difference –11.67, 95% CI –16.99 to –6.36), and the decreases were maintained at T2 (mean difference –11.83, 95% CI –18.19 to –5.46). The HRQoL score in the iMBCR group had significantly larger improvements than that in the control group at T1 and T2 (mean difference 6.66, 95% CI 3.43-9.90 and mean difference 11.94, 95% CI 7.56-16.32, respectively).

**Conclusions:**

Our preliminary findings suggest that the iMBCR program effectively improved the symptom burden and HRQoL of patients with breast cancer, and the participants in the iMBCR group demonstrated good adherence and completion rates. These results indicate that the iMBCR intervention might be a promising way to reduce symptom burden and improve HRQoL of patients with cancer.

**Trial Registration:**

Chinese Clinical Trial Registry ChiCTR2000038980; http://www.chictr.org.cn/showproj.aspx?proj=62659

## Introduction

### Background

Breast cancer has become the most frequent cancer type among women. The International Agency for Research on Cancer has reported that the 5-year prevalence of breast cancer is approximately 7.8 million cases globally [1]. In China, the estimated 5-year prevalence of breast cancer is approximately 1,390,095 cases, with a prevalence rate of nearly 197 per 100,000 [2]. The breast cancer burden has grown over time in China [3].

Cancer diagnosis and treatment are highly stressful experiences that cause patients to experience a range of physical or psychological symptoms; for example, up to 86% of patients with breast cancer report fatigue during treatment [4], 54% to 78% report sleep disturbances [5], 53% experience pain [6], and 70% to 81% report psychological distress [7]. Such symptom burdens negatively affect their health-related quality of life (HRQoL) and increase their need for supportive care service. Recently, a large cohort study evaluating HRQoL in this population demonstrated that the restrictions in HRQoL can persist for >10 years [8]. Evidence suggests that psychosocial interventions such as mindfulness-based interventions (MBIs) are effective in improving stress-related symptoms and HRQoL [9,10]. Mindfulness-based stress reduction (MBSR) is a structured 8-week group program initially designed to reduce chronic pain and stress-related symptoms by developing mindfulness, meaning a nonjudgmental and accepting moment-by-moment awareness [11]. It has been adapted for multiple patient populations, and mindfulness-based cancer recovery (MBCR) is an adaptation of MBSR specifically for patients with cancer [12]. This program is not used for cancer treatment but for stressors related to the disease. MBCR retains the core principles and practices of MBSR, and it adds specific intervention material for coping with cancer, further focusing on symptoms such as sleep problems, pain, and fear of cancer recurrence. Growing evidence supports the MBCR program’s benefit for symptoms in patients with breast cancer; for example, previous MBCR studies have repeatedly shown improvements in fatigue [13,14], and several systematic reviews have summarized the benefits of MBCR and other MBIs across outcomes of sleep disturbance, depression, stress, and HRQoL in patients with breast cancer [15,16].

Although the efficacy of MBIs has been proven, patients with cancer still report some barriers to participation. Eyles et al [17] found that the 8-week commitment to the course is the main reason for nonparticipation. Even for those who participated in the intervention, the completion rate of 8-week courses was low; for example, Carlson et al [14] reported that only 64.9% (87/134) of the patients with breast cancer completed the 8-week course. Another study has identified that the sample attrition rate is high (52%) in the MBCR group [18]. Compared with the 8-week intervention, short-term MBIs may be more acceptable to patients with cancer and could save time and human resources. Many attempts have been made to abbreviate 8-week MBIs [19]; for example, Demarzo et al [20] explored the efficacy of standard 8-week MBIs and abbreviated 4-week MBIs for improving well-being in a nonclinical population. They found a similar efficacy between the 4-week and 8-week MBIs. Similarly, another study by Wirth et al [21] identified that a 4-week MBCR program significantly improved sleep quality among patients with cancer. Both of these studies showed high retention in participants (4-week group attrition rate: 4.2% and 5%, respectively). Thus, the effectiveness of a 4-week MBCR program for patients with breast cancer is worth exploring.

Studies have also identified other practical barriers that may diminish access of patients with cancer to face-to-face programs, including but not limited to cancer-related illnesses, limited mobility, fatigue, transportation inconvenience, and scarcity of trained therapists [22-24]. Moreover, COVID-19 regulations have restricted people’s activities. An internet-delivered intervention might be a promising method to overcome these barriers. Zernicke et al [25] assessed the feasibility of delivering a web-based MBCR program to patients with cancer. Their findings support providing the web-based MBCR program to patients with cancer, that is, feasibility targets for recruitment and retention are achieved, and participants are satisfied with a web-based MBCR program. However, evidence of the effect of an internet-delivered MBCR (iMBCR) program in women with breast cancer is limited. Further research is required to explore the efficacy of an iMBCR program in patients with breast cancer.

### Objectives

In this study, we constructed a 4-week iMBCR program, and the heuristic framework developed by Barrera and Castro [26] guided the process of cross-cultural intervention adaptation [26]. This study aimed to investigate the effectiveness of the 4-week iMBCR program in regard to changes in patients’ symptom burden and HRQoL. We developed the following hypotheses: (1) women allocated to the iMBCR group would report greater reductions in symptom burden and improvements in HRQoL after the intervention and at 1-month follow-up than those allocated to the control group, and (2) a 4-week iMBCR program would perform well in terms of adherence and intervention completion rates.

## Methods

### Participants

Our study used the following inclusion criteria: (1) women aged between 18 and 70 years, (2) having a prior diagnosis with stages 0 to IV breast cancer and aware of their cancer diagnosis, (3) having completed 1 to 24 months after surgery, (4) with normal cognitive capacity and functional status (Mini-Mental State Examination score ≥27 points and Karnofsky performance status scale score >60 points), and (5) able to operate a smartphone and WeChat (the most popular smartphone app used for communication in China). The exclusion criteria were as follows: (1) participating in other psychological interventions or consultations, (2) having a history of mental illness or a combination of other severe somatic diseases, and (3) refusing to participate.

Sample size was calculated by power analyses using G*Power software (version 3.1; Heinrich Heine University). According to a previous study that explored the effectiveness of the MBCR program in women with cancer, the effect size for HRQoL scores was 0.66 [27]. Thus, to predict the difference between the 2 groups at a 5% level of significance and a power of 0.8, 38 participants were required in each group. Allowing for a 20% attrition rate, an additional 10 participants were needed in each group. Thus, the total sample size required for this study was 96, with 48 participants in each group.

### Study Design and Randomization

This study was a 2-arm, parallel-group randomized controlled trial. Randomization was performed after the participants agreed to participate and signed the informed consent. Patients were randomly divided into the iMBCR group and the control group in a 1:1 ratio according to a list of computer-generated random numbers. To guarantee allocation concealment, an independent researcher who was not involved in the recruitment performed the random assignments by delivering an opaque, sealed envelope to each participant. Because of the nature of the intervention, participants could not be blinded. The research assistants who collected the data were blinded to each participant’s group allocation throughout the study.

### Ethics Approval

This study was approved by the institutional review board of the Xiangya School of Nursing (E2020153), Central South University, and was registered at the Chinese Clinical Trial Registry (ChiCTR2000038980).

### Recruitment and Data Collection

Participants were recruited at the breast cancer wards of a tertiary hospital in Changsha, Hunan province, China, between November 1, 2020, and August 15, 2021. Recruitment was conducted primarily through referrals from ward nurses and research posters displayed at the gynecological clinic and wards. We contacted interested participants to screen them for eligibility. During this time, 1 researcher used the Mini-Mental State Examination [28] and Karnofsky performance status scale [29] to assess each participant’s cognitive and functional status. Next, we contacted the eligible participants and provided further information about our study to them. Participants were given the choice to participate or decline and were informed that they had the right to withdraw at any time without reprisal. Baseline data were collected using written questionnaires at the wards of the hospitals. Postintervention and 1-month follow-up results were collected through web-based questionnaires. Participants who attended <2 sessions were considered dropouts.

### Intervention

#### iMBCR Group

Guided by the heuristic framework developed by Barrera and Castro [26], the original English version of the MBCR program was translated into Chinese with a cross-cultural adaptation process. We used the following steps:

1. Information gathering: the goal of this step is to identify the form and content of needed adaptations, as well as the characteristics and preferences of potential participants. We conducted a mixed study. The quantitative study investigated the supportive care needs, mindfulness levels, and HRQoL of Chinese patients with cancer. The results showed that patients with cancer had a high level of supportive care needs. Health system and information needs and psychological needs were the top 2 needs of Chinese patients with cancer. Multiple linear regression analyses revealed that psychological needs and mindfulness levels could significantly predict HRQoL in patients with cancer. This suggested that the HRQoL of patients with cancer might be improved with mindfulness-based psychological interventions. The qualitative study was conducted to understand fully the patients’ cancer-related troubles or distress or discomfort, what they thought were the main reasons for these issues, and their attitudes toward participating in an 8-week psychosocial intervention. The interviews showed that patients with cancer experienced numerous symptoms but lacked strategies for coping with them. In addition, most (6/10, 60%) of the patients felt that an 8-week intervention course was too long for them.
2. Preliminary adaptation design: we translated the text of the 8-week MBCR program into Chinese and organized an expert-panel meeting to discuss the content and delivery format of an MBCR program in the Chinese cultural context. Combining the results of the previous phase with those of the expert-panel meeting, we created the preliminary 8-week web-based MBCR program.
3. Preliminary adaptation tests: we conducted a pilot study to test the feasibility and acceptability of an 8-week iMBCR program in patients with breast cancer. The results showed that the web-based MBCR intervention was acceptable to the participants, but only 40% (4/10) of the participants completed the full 8-week session. More than half (7/10, 70%) of the participants felt that the intervention was too long. Interviews with the participants who completed the intervention (4/10, 40%) showed that mindful practice techniques (eg, mindful breathing, body scan meditation, and mindful walking) were practical for daily life activities, but yoga exercises were difficult for some (3/4, 75%) of the patients to complete.
4. Adaptation refinement: on the basis of the problems in the pilot study and participant feedback, we adjusted the duration and content of the iMBCR program. Five experts were invited to evaluate the content importance and rationality of a 4-week MBCR program. Through 2 rounds of expert consultations, we constructed the 4-week iMBCR program. Table 1 presents the detailed contents of the 4-week iMBCR program.

**Table 1:** Content of the 4-week mindfulness-based cancer recovery program.

Sessions and modules		Content
**Session 1: first experience of mindfulness—connecting mind and body**		
	Participative section	Self-introduction; review of the agenda and content of sessions and main rules; group discussion on changes and distress caused by cancer
	Didactic presentation	Understand the core concepts and related knowledge of mindfulness; develop a mindful attitude
	Mindfulness techniques	Raisin meditation; body scan practice
	Home practice	Guided audio body scan practice; mindful eating; record pleasant and unpleasant events
**Session 2: power of awareness—emotion and thought**		
	Participative section	Recording pleasant and unpleasant events; mindfulness practice experience and body reaction
	Didactic presentation	Explain nonjudgmental attitude; self-acceptance of emotions and thoughts
	Mindfulness techniques	Mindful breathing; mindful stretching; sitting meditation
	Home practice	Guided audio sitting meditation; mindful breathing; observe individual responses to stressful events
**Session 3: stress management and self-compassion**		
	Participative section	Group discussion: individual responses to stressful events; confusion or discovery in practice
	Didactic presentation	Mindfulness coping with cancer-related symptoms and self-compassion
	Mindfulness techniques	Mindful walking; loving-kindness meditation; sitting meditation
	Home practice	Guided audio body scan practice; mindful walking; loving-kindness meditation
**Session 4: new life—incorporating mindfulness into daily life**		
	Participative section	How to bring mindfulness into daily living
	Didactic presentation	Attitude toward mindfulness practices; experience sharing; recommendation of resources for mindfulness practice
	Mindfulness techniques	“Who am I?” meditation exercise; mindful breathing; mindful stretching
	Home practice	Guided audio sitting meditation; loving-kindness meditation

The iMBCR group received a 4-week MBCR program (1.5 hours per week) and at least 30 minutes of daily mindfulness home practice. All participants were invited to scan a quick-response code to join a WeChat group. We used this group mainly to send to the participants links to web-based courses and intervention materials, as well as for instant web-based communication. Participants were invited to attend web-based courses on Saturday mornings through a videoconferencing app (Tencent) extensively used in China. They were required to use their own smartphone to access the web-based course, which was also accessible by a desktop computer. Our assistant provided systematic training on the use of videoconferencing to ensure that the courses were accessible to participants. Session attendance was recorded, and all courses were recorded by video. The course video was provided to those who were absent for any reason. A Chinese-version MBCR book and some assisted–mindfulness practice audios were provided to the participants for home practice. Participants were asked to use the WeChat applet to make a note after completing their daily home practice. The intervention was delivered by a therapist who had completed mindfulness training and had 4 years of experience in teaching MBCR, accompanied by an assistant with 2 years of experience in mindfulness practice.

#### Control Group

Participants in the control group received usual care (routine standard oncology care) and 4 cancer-themed health education sessions: recognizing stress and managing negative emotions, coping with the adverse effects of therapy, dietary guidance, and exercise guidance, which do not involve any mindfulness component. These 4 sessions were conducted through Tencent. The number and frequency of web-based courses were the same as those in the iMBCR group.

### Treatment Fidelity

Several strategies were used to ensure treatment fidelity. A treatment manual specifying the content of each course was developed, and the interventions were followed strictly. All of the intervention courses were video recorded, and an investigator reviewed the video recording after each course to ensure proper implementation of the treatment manual. Furthermore, our team met at the end of each intervention week to discuss the intervention implementation quality.

### Measures

#### Demographic and Clinical Characteristics

A self-designed demographic questionnaire was used to collect the demographic data of the participants, including age, education, marital status, employment status, religion, and meditation and yoga practice experience. Clinical information included time since diagnosis, stage of breast cancer, and type of treatment.

#### Symptom Burden

The Chinese version of the MD Anderson Symptom Inventory (MDASI-C) was used to evaluate the severity of symptoms and symptom interference with daily life [30]. We used the MDASI-C to evaluate the symptom burden of women diagnosed with breast cancer. The MDASI-C includes 13 core symptom-severity items (pain, fatigue, sleep disturbance, drowsiness, lack of appetite, nausea, vomiting, shortness of breath, numbness, difficulty remembering, dry mouth, distress, and sadness) and 6 symptom-interference items (general activity, walking, work, mood, relations with other people, and enjoyment of life). Each item was measured using a numeric rating scale ranging from 0 (not present) to 10 (as bad as I can imagine) to evaluate the participants’ status over the past 24 hours. The internal consistency for the MDASI-C ranged from Cronbach α=.84 to Cronbach α=.90 [31].

#### HRQoL of Women Diagnosed With Breast Cancer

The Chinese version of the Functional Assessment of Cancer Therapy-Breast (FACT-B) scale was used to assess the HRQoL of women diagnosed with breast cancer. This is a 36-item questionnaire that includes the 27 items of general HRQoL associated with cancer and the 9 items of HRQoL related to breast cancer. FACT-B comprises the following subscale domains: physical well-being, social well-being, emotional well-being, functional well-being, and breast cancer–specific subscale. Each item was measured using a numeric rating scale ranging from 0 (not at all) to 4 (very much) to evaluate the participants’ status over the past week. A higher score indicates better HRQoL. The test-retest reliability of the Chinese version of the FACT-B scale ranged from .82 to .89, and the Cronbach α coefficient ranged from .61 to .84 [32].

#### Intervention Adherence and Completion

Adherence to the intervention was calculated by dividing the number of performed training sessions by the number of recommended training sessions. The 4-week iMBCR program corresponded with 4 web-based sessions and 28 days of home practice. Completion was estimated by dividing the number of consenting participants by the number of participants who completed the study.

### Statistical Methods

Data were analyzed using SPSS software (version 18.0; IBM Corp). The intention-to-treat analysis was applied. Missing data (<20%) were handled with the participants’ average response on the remaining scale items, and the missing data in the FACT-B questionnaire were treated by the proportion method per the manual instructions [33]. Descriptive statistics were applied to calculate the mean and SD for continuous data and frequency and percentage for categorical data. Baseline differences between the groups were explored using a chi-square test or Fisher exact test for categorical variables and a 2-tailed independent sample *t* test for continuous variables. Linear mixed models were used to compare the groups over time on all outcome variables. The data were hierarchically arranged in a 2-level structure with time at level 1 nested within individuals at level 2. Fixed effects were specified for intercept, time, group, and the group×time interaction, whereas the random effect was the participant. Effect sizes for the mean changes between the groups were calculated using Cohen *d*, with 0.2, 0.5, and 0.8 considered a small effect size, medium effect size, and large effect size, respectively [34]. We assumed a 2-tailed *P* value of <.05 to be statistically significant.

## Results

### Participant Characteristics

A total of 103 participants were recruited; 51 (49.5%) participants in the iMBCR group and 52 (50.5%) participants in the control group. The age of the participants ranged from 28 to 67 (average 46.8, SD 7.9) years. The median length of time since diagnosis was 4 months. The majority (86/103, 83.5%) of the patients were diagnosed with stage II or stage III breast cancer. No significant differences were detected in the baseline characteristics between the iMBCR and control groups (none of the *P* values met the threshold for statistical significance). Details are listed in Table 2.

**Table 2:** Sociodemographic and clinical characteristics of the participants (N=103).

Characteristics		iMBCR^a^ group (n=51)		Control group (n=52)	*t* test (*df*) or chi-square value (*df*)	*P* value	
Age (years), mean (SD; range)		45.37 (7.59; 28-67)		48.17 (8.05; 29-64)	*t* (*df*)=–1.816 (101)	.07	
**Education, n (%)**					*χ*^2^ (*df*)=4.798 (3)		.19
	College or university	18 (35)		9 (17)			
	High school or vocational	18 (35)		21 (40)			
	Secondary	9 (18)		15 (29)			
	≤Primary	6 (12)		7 (14)			
**Marital status, n (%)**					*χ*^2^ (*df*)=0.000 (1)		>.99
	Married	49 (96)		49 (94)			
	Other marital status	2 (4)		3 (6)			
**Employment status, n (%)**					*χ*^2^ (*df*)=0.816 (2)		.67
	Employed	30 (59)		30 (58)			
	Unemployed	16 (31)		14 (27)			
	Retired	5 (10)		8 (15)			
**Religion, n (%)**					*χ*^2^ (*df*)=1.414 (1)		.23
	No	48 (94)		52 (100)			
	Yes	3 (6)		0 (0)			
**Experience of meditation practice, n (%)**					*χ*^2^ (*df*)=0.344 (1)		.56
	No	42 (82)		45 (87)			
	Yes	9 (18)		7 (13)			
**Experience of yoga practice, n (%)**					*χ*^2^ (*df*)=0.079 (1)		.78
	No	36 (71)		38 (73)			
	Yes	15 (29)		14 (27)			
**Time since diagnosis, months, n (%)**					*χ*^2^ (*df*)=3.945 (2)		.14
	≤3	25 (49)		24 (46)			
	4 to 12	15 (29)		23 (44)			
	≥13	11 (22)		5 (10)			
**Stage of breast cancer, n (%)**					*χ*^2^ (*df*)=6.728 (4)		.13
	0 (carcinoma in situ)	2 (4)		1 (2)			
	I	6 (12)		2 (4)			
	II	28 (55)		31 (59)			
	III	10 (19)		17 (33)			
	IV	5 (10)		1 (2)			
**Treatment, n (%)**							
	Breast surgery	51 (100)		52 (100)	N/A^b^	N/A	
	Chemotherapy	48 (94)		50 (96)	*χ*^2^ (*df*)=0.000 (1)	.98	
	Radiotherapy	16 (31)		12 (23)	*χ*^2^ (*df*)=0.895 (1)	.34	
	Immunotherapy	14 (27)		20 (38)	*χ*^2^ (*df*)=1.412 (1)	.24	
**Baseline psychometric scores, mean (SD)**							
	MDASI^c^	63.12 (20.37)	61.44 (19.58)		*t* (*df*)=0.427 (101)	.67	
	FACT-B^d^	82.64 (15.74)	82.86 (16.27)		*t* (*df*)=−0.070 (101)	.95	

^a^iMBCR: internet-delivered mindfulness-based cancer recovery.

^b^N/A: not applicable.

^c^MDASI: MD Anderson Symptom Inventory.

^d^FACT-B: Functional Assessment of Cancer Therapy-Breast.

### Intervention Adherence and Completion

**Figure 1:**
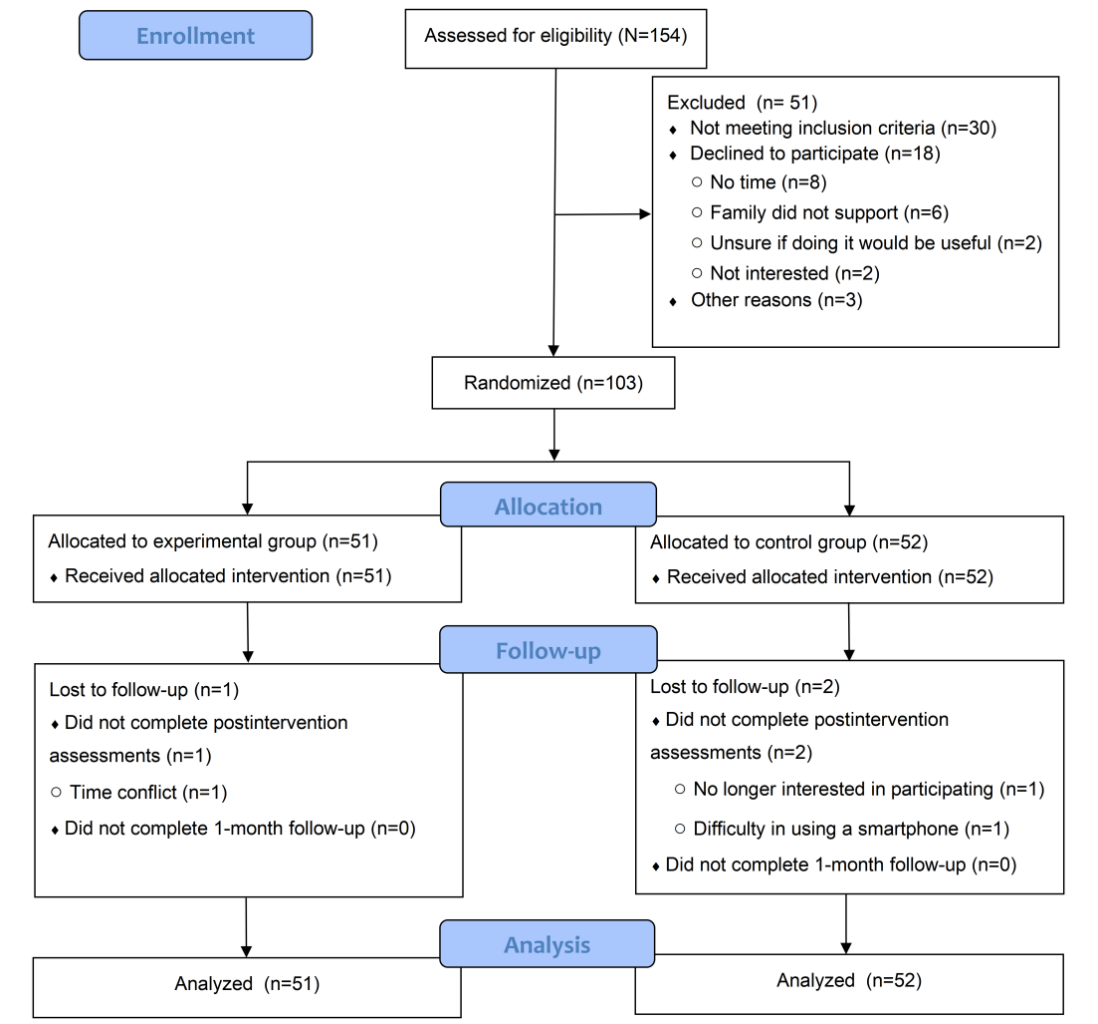
CONSORT (Consolidated Standards of Reporting Trials) flow diagram.

Figure 1 shows the flowchart of participants throughout the study. A total of 100 participants completed all assessments, of whom 1 (1%) participant was lost from the iMBCR group (dropout rate=1/51, 2%), and 2 (2%) were lost from the control group (dropout rate=2/52, 4%). No significant differences were detected between the lost sample and the sample that completed all assessments (none of the *P* values met the threshold for statistical significance). The mean number of attended iMBCR courses was 3.6 (SD 0.7; adherence rate=3.6/4, 90%). The mean number of days of mindfulness home practice was 19.6 (SD 4.6; adherence rate=19.6/28, 70%).

### Effect on Outcomes

#### Symptom Burden

Statistically significant group×time effects were observed for MDASI total score (*F*_2,100_=9.86; *P*<.001) and the symptom-severity subscale (*F*_2,100_=11.73; *P*<.001) between the iMBCR group and control group, indicating that the MDASI total score and symptom-severity scores in both groups had different trends over the 3 time points (Table 3). Compared with the participants in the control group, those in the iMBCR group had significantly larger decreases in the MDASI total score (mean difference –11.67, 95% CI –16.99 to –6.36, Cohen *d*=–0.65) and symptom-severity scores (mean difference –8.87, 95% CI –12.54 to –5.21, Cohen *d*=–0.69) at T1, and the difference remained significant at T2 (mean difference –11.83, 95% CI –18.19 to –5.46, Cohen *d*=–0.98 and mean difference –9.51, 95% CI –14.19 to –4.82, Cohen *d*=–0.96, respectively). Table 4 shows the details of mean symptom-severity scores. Sleep disturbance, fatigue, and pain were the 3 most severe physical symptoms in both groups at the baseline. Regarding the between-group comparisons, fatigue was significantly decreased in the iMBCR group at T1 and T2 (none of the *P* values met the threshold for statistical significance), and sleep disturbance only significantly decreased at T1 (*P=*.02). The most severe psychological symptoms were sadness and distress, and only distress had significantly decreased at T1 (*P=*.01) in the iMBCR group compared with the control group.

The group×time interaction for the symptom-interference subscale was not significant (*P*=.26). Reductions were also observed in symptom-interference scores at T1 and T2, but the difference did not reach statistical significance.

**Table 3:** Comparison of symptom burden and health-related quality of life in the experimental and control groups.

Scale		iMBCR^a^ group, mean (SD)	Control group, mean (SD)	Cohen *d*		Linear mixed model statistical tests																																
						Difference in mean change from baseline between groups^b^					Group effect							Time effect											Group×time									
						Score (95% Cl)		*P* value			*F* test (*df*)			*P* value				*F* test (*df*)							*P* value			*F* test (*df*)							*P* value			
**MDASI^c^ total score**												5.55 (1,95)				.02				65.31 (2,100)						<.001				9.86 (2,100)						<.001		
	T0^d^	63.12 (20.37)	61.44 (19.58)	N/A^e^		N/A		N/A																														
	T1^f^	45.11 (13.30)	54.78 (16.46)	–0.65		–11.67 (–16.99 to –6.36)		<.001																														
	T2^g^	39.14 (9.95)	49.13 (10.43)	–0.98		–11.83 (–18.19 to –5.46)		<.001																														
**MDASI symptom-severity score**												4.91 (1,102)				.03				69.61 (2,100)						<.001				11.73 (2,100)						<.001		
	T0	45.43 (13.79)	43.35 (14.74)	N/A		N/A		N/A																														
	T1	31.87 (8.85)	38.53 (10.43)	–0.69		–8.87 (–12.54 to –5.21)		<.001																														
	T2	26.92 (7.49)	34.27 (7.79)	–0.96		–9.51 (–14.19 to –4.82)		<.001																														
**MDASI symptom-interference score**												4.56 (1,161)				.03				20.81 (2,121)						<.001				1.37 (2,121)						.26		
	T0	17.69 (9.78)	18.10 (7.24)	N/A		N/A		N/A																														
	T1	13.23 (6.41)	16.24 (7.30)	–0.44		–2.67 (–6.14 to 0.79)		.13																														
	T2	12.22 (4.20)	14.86 (3.68)	–0.67		–2.30 (–5.14 to 0.54)		.11																														
**FACT-B^h^ total score**												6.55 (1,96)				.01				36.49 (2,100)						<.001				14.82 (2,100)						<.001		
	T0	82.64 (15.74)	82.86 (16.27)	N/A		N/A		N/A																														
	T1	90.07 (13.74)	84.04 (11.52)	0.48		6.66 (3.43 to 9.90)		<.001																														
	T2	98.00 (7.51)	86.52 (10.88)	1.23		11.94 (7.56 to 16.32)		<.001																														
**Physical well-being**												1.88 (1,101)				.17				20.48 (2,99)				<.001						5.18 (2,99)				.007				
	T0	19.41 (4.23)	19.63 (5.41)	N/A		N/A		N/A																														
	T1	21.38 (3.60)	20.20 (4.66)	0.28		1.50 (0.38 to 2.62)		.009																														
	T2	22.88 (2.02)	20.86 (4.76)	0.55		2.31 (0.87 to 3.76)		.002																														
**Emotional well-being**												0.41 (1,101)				.53				25.42 (2,96)				<.001						2.20 (2,96)				.12				
	T0	15.20 (4.81)	15.35 (4.34)	N/A		N/A		N/A																														
	T1	16.46 (3.58)	16.32 (3.97)	0.04		0.35 (–0.66 to 1.36)		.50																														
	T2	18.46 (1.95)	17.26 (3.59)	0.42	1.38 (–0.05 to 2.81)		.06																															
**Functional well-being**										3.22 (1,102)					.08				5.25 (2,90)				.007						4.89 (2,90)				.01					
	T0	11.73 (4.74)	11.66 (4.77)	N/A	N/A		N/A																															
	T1	12.36 (4.78)	10.10 (4.36)	0.49	1.31 (0.14 to 2.48)		.03																															
	T2	14.06 (2.68)	11.48 (4.32)	0.72	2.52 (0.90 to 4.15)		.003																															
**Social** **well-being**										2.33 (1,101)					.13				2.02 (2,96)				.14						1.51 (2,96)				.23					
	T0	17.19 (5.31)	16.86 (5.21)	N/A	N/A		N/A																															
	T1	17.87 (4.84)	16.58 (3.70)	0.30	1.03 (–0.23 to 2.29)		.11																															
	T2	18.69 (3.03)	16.98 (3.58)	0.52	1.41 (–0.38 to 3.21)		.12																															
**Breast cancer–specific subscale for additional concerns**										33.41 (1,99)					<.001				46.72 (2,196)				<.001						29.33 (2,196)									<.001
	T0	19.12 (2.42)	19.37 (2.39)	N/A	N/A		N/A																															
	T1	22.00 (2.37)	19.94 (2.33)	0.88	2.36 (1.26 to 3.47)		<.001																															
	T2	23.92 (2.16)	19.94 (2.48)	1.70	4.28 (3.18 to 5.39)		<.001																															

^a^iMBCR: internet-delivered mindfulness-based cancer recovery.

^b^Difference in mean change from baseline to end point between the groups.

^c^MDASI: MD Anderson Symptom Inventory.

^d^T0: baseline.

^e^N/A: not applicable.

^f^T1: after the intervention.

^g^T2: 1-month follow-up.

^h^FACT-B: Functional Assessment of Cancer Therapy-Breast.

**Table 4:** Details of changes in symptom severity.

Symptom	Severity rank	Symptom severity									Difference in mean change between groups^a^				
		iMBCR^b^ group, mean (SD)				Control group, mean (SD)				T0-T1				T0-T2	
		T0^c^	T1^d^	T2^e^	T0		T1	T2	Score (95% Cl)			*P* value	Score (95% Cl)		*P* value
Sleep disturbance	1	4.8 (2.1)	3.6 (1.4)	3.0 (1.0)	4.7 (2.5)		4.3 (1.9)	3.6 (1.2)	–0.81 (–1.46 to –0.16)			.02	–0.71 (–1.52 to 0.09)		.08
Sadness	2	4.6 (2.2)	3.1 (1.3)	2.6 (0.9)	4.2 (2.2)		3.5 (1.8)	2.8 (1.1)	–0.87 (–1.76 to 0.019)			.06	–0.68 (–1.46 to 0.09)		.08
Distress	3	4.3 (2.1)	3.0 (1.2)	2.8 (1.0)	4.3 (2.1)		3.8 (1.7)	3.3 (1.2)	–0.72 (–1.29 to –0.16)			.01	–0.52 (–1.26 to 0.22)		.17
Fatigue	4	4.2 (1.9)	2.9 (1.3)	2.6 (1.1)	3.8 (1.9)		3.3 (1.5)	2.8 (1.1)	–0.92 (–1.62 to –0.21)			.01	–0.69 (–1.36 to –0.02)		.04
Pain	5	3.6 (2.4)	2.6 (1.3)	2.2 (1.1)	3.9 (2.3)		3.2 (1.5)	3.1 (1.0)	–0.28 (–0.92 to 0.37)			.40	–0.58 (–1.43 to 0.27)		.18
Drowsiness	6	3.9 (2.3)	2.5 (1.4)	1.9 (0.9)	3.4 (1.6)		3.0 (1.4)	2.8 (1.1)	–0.95 (–1.66 to –0.25)			.009	–1.10 (–1.76 to –0.44)		<.001
Lack of appetite	7	3.6 (2.3)	2.3 (1.3)	1.6 (0.7)	3.3 (2.1)		3.0 (1.4)	2.4 (1.0)	–1.07 (–1.71 to –0.43)			<.001	–1.18 (–2.01 to –0.35)		.006
Dry mouth	8	3.4 (1.8)	3.0 (1.6)	2.5 (1.1)	3.3 (1.9)		3.4 (1.4)	3.1 (1.1)	–0.68 (–1.25 to –0.12)			.02	–1.07 (–1.76 to –0.37)		.003
Difficulty remembering	9	3.2 (2.4)	2.4 (1.6)	1.9 (1.1)	3.4 (2.2)		3.2 (1.8)	2.8 (1.4)	–0.65 (–1.18 to –0.09)			.02	–0.81 (–1.61 to –0.01)		.048
Numbness	10	2.9 (1.8)	2.1 (1.2)	1.9 (0.8)	3.2 (1.9)		2.7 (1.6)	2.2 (1.1)	–0.34 (–1.11 to 0.44)			.14	–0.02 (–0.71 to 0.67)		.04
Nausea	11	2.7 (2.1)	1.8 (1.3)	1.6 (0.9)	2.6 (1.9)		2.4 (1.6)	2.3 (0.9)	–0.76 (–1.28 to –0.24)			.005	–0.85(–1.62 to –0.08)		.03
Vomiting	12	2.3 (2.1)	1.4 (0.8)	1.1 (0.5)	2.1 (1.5)		1.8 (1.4)	1.7 (0.8)	–0.58 (–1.36 to 0.19)			.39	–0.74 (–1.43 to –0.05)		.96
Shortness of breath	13	1.8 (1.3)	1.2 (0.9)	1.2 (0.6)	1.3 (1.4)		0.9 (1.1)	1.2 (0.7)	–0.24 (–0.64 to 0.15)			.23	–0.57 (–1.11 to –0.03)		.04

^a^Difference in mean change from baseline to end point between the groups.

^b^iMBCR: internet-delivered mindfulness-based cancer recovery.

^c^T0: baseline.

^d^T1: after the intervention.

^e^T2: 1-month follow-up.

#### HRQoL Assessment

The results indicated a significant group×time interaction for the FACT-B total score (*F*_2,100_=14.82; *P*<.001), physical well-being (*F*_2,99_=5.18; *P*=.007), functional well-being (*F*_2,90_=4.89; *P*=.01), and the breast cancer–specific subscale (*F*_2,196_=29.33; *P*<.001). Participants in the iMBCR group had larger improvements in the FACT-B total score at T1 and T2 than the control group (mean difference 6.66, 95% CI 3.43-9.90, Cohen *d*=0.48 and mean difference 11.94, 95% CI 7.56-16.32, Cohen *d*=1.23, respectively). Statistical improvements were also observed in physical well-being, functional well-being, and the breast cancer–specific subscale at T1 and T2. Emotional well-being exhibited only a time-based effect (*F*_2,96_=25.42; *P*<.001), and the difference in mean change between groups was not significant for T1 and T2. For social well-being, we found no significant group, time, or group×time interaction effects, indicating that the effect of the iMBCR program on patients’ social well-being was not significant.

## Discussion

### Principal Findings

To our knowledge, this study is the first randomized controlled trial to investigate the effect of an iMBCR program in Chinese women diagnosed with breast cancer and adds to the few available studies on short-term MBIs. We found that the outcomes of symptom burden and HRQoL were improved immediately after the intervention, and the effect was maintained at the 1-month follow-up. This finding demonstrated that the 4-week iMBCR program is effective for women with breast cancer. Moreover, our intervention demonstrated good adherence and intervention completion rates.

Our study found that the MDASI total score in the iMBCR group had significantly decreased after the intervention and at 1-month follow-up compared with the control group. The findings supported our first hypothesis that the 4-week iMBCR program can relieve the symptom burden of women diagnosed with breast cancer. The results of reduced symptom burden after the intervention were consistent with a previous face-to-face 6-week group MBSR study in patients with breast cancer [35], which may indicate that the web-based mindfulness intervention may have an effect on symptoms that is similar to the effect of face-to-face interventions. The symptom-severity scores in the iMBCR group significantly decreased after the intervention. However, the effect was stronger at 1-month follow-up. The reasons may be that the severity of symptoms decreased over time and that the 4-week iMBCR intervention activated the patients’ mindfulness behavior in daily life. Techniques taught in iMBCR courses, such as mindful breathing, body scan meditation, mindful stretching, and sitting meditation, had been internalized by the patients and could be useful when they experience symptoms or stressful events in daily life.

Consistent with existing symptom research on patients with breast cancer, we found that the top 3 severe physical symptoms at baseline were fatigue, sleep disturbance, and pain [36]. Current systematic reviews have concluded that standard 8-week MBIs could improve fatigue and quality of sleep in short-term (end of intervention) to medium-term (up to 6 months after baseline) effects for women diagnosed with breast cancer [15,37]. Regarding fatigue, our study found that the 4-week iMBCR intervention achieved results that were similar to those of previous studies. However, we did not find significant reductions in sleep disturbance during follow-up, which may be due to the fact that the 4-week iMBCR program was not intended to treat sleep problems and that sleep disturbance was assessed only by a single item instead of a standard sleep scale. Similar to previous study results [38,39], we did not find any significant effects of the iMBCR intervention on pain in patients with breast cancer.

As expected, the FACT-B total scores and most domain scores (physical well-being, function well-being, and breast cancer–specific additional concerns) significantly increased among the iMBCR group compared with the control group after the intervention and at 1-month follow-up, indicating that the 4-week iMBCR program could improve patients’ HRQoL. The postintervention effect sizes for HRQoL found in our study (Cohen *d*=0.48) were within the same range as that in a previous study on a face-to-face standard 8-week group MBSR intervention for women diagnosed with stages 0 to III breast cancer (Cohen *d*=0.60) [40]. This finding suggests that the 4-week iMBCR intervention could achieve an effect on HRQoL that is similar to that achieved by standard 8-week MBIs. These results may be caused by multiple reasons. First, the iMBCR program in this study developed the patients’ capacity for the intentional self-regulation of attention as well as the attitude and practice of acceptance, which has been proven to be effective in reducing negative reactivity and improving stress-related health outcomes [41]. Meanwhile, the improvement in the patients’ symptom burden positively affected the HRQoL [42]. Besides, the group intervention format provided a path of communication and emotional support for patients with breast cancer. Social support reportedly predicted better adjustment to cancer and better quality of life [43].

Unlike previous studies, we found no intervention effect on emotional well-being [39], which was also reflected in the results of symptom severity (no intervention effect on sadness, and distress significantly reduced only during the intervention). The results of another 4-week MBCR program study also showed no positive intervention effects on psychological outcomes (depression and perceived stress) [21]. It seemed that a short-term mindfulness intervention was more effective for physical well-being than for emotional well-being, and the improvement in psychological outcomes required a longer mindfulness intervention period. Nevertheless, given the relative scarcity of studies on short-term mindfulness interventions, we were limited in our ability to draw any conclusion in this regard.

In this study, the attrition rate among the participants in the iMBCR group (dropout rate=1/51, 2%) was relatively low, and it was lower than that reported in the standard 8-week MBIs (typically 20%-30%) [44]. Furthermore, the adherence rate was high in both iMBCR courses and mindfulness home practice. These findings confirmed our second hypothesis. Our results demonstrated that the 4-week mindfulness intervention is acceptable for patients with breast cancer.

### Limitations

This study includes several limitations. First, the completion of mindfulness home practice relied on the self-reporting of participants and was calculated by days and not specific practice minutes. This setup may affect the accuracy of intervention adherence data because self-reporting is subjective. In a future study, we will consider using more advanced technology to record user log-ins or the time spent on the web to measure intervention completion and adherence rates. Second, the heterogeneity of the sample with regard to cancer stage and treatment type may affect symptom burden and HRQoL. However, the differences in the cancer stages and treatment types that existed between the 2 groups at baseline were not statistically significant, which could have reduced the bias to some extent. Third, the patients in this study were followed up for only 1 month. We were unable to determine the medium- or long-term effects of the 4-week iMBCR program on symptom burden and HRQoL. As such, future research should incorporate longer follow-up periods to examine the durability of the effects of an iMBCR program. Fourth, our participants were aged <70 years, and it is unclear whether the program is feasible for, or would benefit, patients aged >70 years. Finally, because the study was conducted on the web, patients who did not own technological devices may have lost the opportunity to participate in the study.

### Research Implications

Our study used a relatively abbreviated intervention and a novel technology that made the MBCR program more acceptable for patients with breast cancer. The results were encouraging for implementing this 4-week web-based mindfulness intervention to reduce symptom burden and improve HRQoL of patients with cancer. However, because of the short follow-up period in this study, a long-term follow-up study is required to confirm the results more precisely. Our findings also provided evidence for eHealth services for patients with breast cancer and the effect of short-term MBIs. Future research is warranted to continue the investigation on the mechanisms of change in short-term web-based MBCR interventions. Researchers should examine the efficacy of the components of mindfulness and emphasize not only the techniques but also to what extent they are effective. They should also determine how much exposure to the intervention is needed to efficiently develop a feasible and effective program that increases its accessibility for a larger number of clinical populations.

### Conclusions

Our study explored the effect of a 4-week iMBCR program in patients with breast cancer. The 4-week iMBCR program showed positive effects for symptom burden and HRQoL immediately after the intervention and at 1-month follow-up, and our intervention also demonstrated good adherence and completion rates. This low-cost web-based intervention can be more acceptable for patients and be easily translated into clinical practice to reach numerous patients. Further studies are warranted to explore the long-term effects of, and mechanisms of change in, short-term web-based MBCR interventions.

## Appendix

Multimedia Appendix 1 CONSORT-eHEALTH (V 1.6.1)

## Abbreviations

FACT-B: Functional Assessment of Cancer Therapy-Breast
HRQoL: health-related quality of life
iMBCR: internet-delivered mindfulness-based cancer recovery
MBCR: mindfulness-based cancer recovery
MBI: mindfulness-based intervention
MBSR: mindfulness-based stress reduction
MDASI: MD Anderson Symptom Inventory
MDASI-C: MD Anderson Symptom Inventory, Chinese version
